# Effect of Ultra-Rapid Heating/Cooling on the Microstructure and Properties of TC4-B-Si Titanium Matrix Composites

**DOI:** 10.3390/ma18184223

**Published:** 2025-09-09

**Authors:** Xiaonan Lu, Jianchao Li, Cheng Liu, Likun Wang, Sainan Ma, Bo Yuan, Bowen Gong, Wenting Ouyang, Huan Wang, Xiang Gao, Huiping Tang, Hua-Xin Peng

**Affiliations:** 1 Ningbo Global Innovation Center, Zhejiang University, Ningbo 315100, China; xnlu@zju.edu.cn (X.L.); wanglk01@zju.edu.cn (L.W.); sainanma@zju.edu.cn (S.M.); 2 Institute for Composites Science Innovation (InCSI), School of Materials Science and Engineering, Zhejiang University, Hangzhou 300027, China; lijianchao@zju.edu.cn (J.L.); 0624448@zju.edu.cn (C.L.); 11726046@zju.edu.cn (B.G.); 12126008@zju.edu.cn (W.O.); hwang2014@zju.edu.cn (H.W.); 3 Institute of Intelligent Manufacturing Technology, ShenZhen Polytechnic University, Shenzhen 518055, China; yuanbo@szpu.edu.cn; 4 Advanced Materials Additive Manufacturing Innovation Research Center, Hangzhou City University, Hangzhou 310015, China; thpz@hzcu.edu.cn

**Keywords:** TC4-B-Si, ultra-rapid cooling, nanocomposite, hardness

## Abstract

Titanium matrix composites (TMCs) possess excellent properties, which are widely applied in various high-end fields. An ultrafine multi-scale network structure may further enhance the TMCs. Then, the application potential is widened. Here, the in situ synthesized TC4-B-Si composites were prepared by selective laser melting technology, to achieve ultrafine microstructure by inducing ultra-rapid heating/cooling process. The preparation process–structure–performance relationships were investigated. It was found that an appropriate laser energy density leads to high-density TMCs with stable molten pools and good interlayer bonding. With the decreasing energy density, the in situ generated TiB network structure is refined from the sub-micron scale to the nano-scale. The most Si atoms are supersaturated solid-dissolved in the titanium matrix. In addition, the TiB distribution becomes heterogeneous. Due to the co-effect of grain refinement and reinforcement distribution, the microhardness shows a rising and then falling trend, with decreasing energy density. With a good balance of these two factors, the maximum value of microhardness reaches 454 HV. Therefore, controlling process parameters is a feasible way to achieve multi-structures, and thus enhanced properties. This method is expected to be used on various lightweight and wear-resistant structural components.

## 1. Introduction

Titanium and its alloys feature low density, high specific strength [1,2], excellent corrosion resistance [3,4], and good biocompatibility [5]. However, the insufficient wear resistance and relatively low hardness of titanium alloys restricted their applications in wear-critical components, e.g., lightweight gears and bearing systems [6,7,8]. In recent years, the titanium matrix composites (TMCs) have shown promise in improving the hardness and overall performance of the matrix alloy, via introducing second-phase particles [9,10,11]. These composites exhibit broad application prospects in aerospace, automotive manufacturing, shipbuilding, and biomedical fields, and have attracted extensive attention [12,13,14,15].

The key design concept of TMCs is to introduce reinforcement into the titanium matrix to achieve high-performance [16]. The in situ synthesized TiB whiskers and Ti_5_Si_3_ particles present strong interfacial bonding with titanium alloy [17,18]. Moreover, their density is low (similar to titanium matrix). Furthermore, the matched thermal expansion coefficient with titanium is significant, which reduces thermal mismatch [19]. As such, the TiB whiskers and Ti_5_Si_3_ particles are preferred for applications in titanium alloy enhancement. Due to different solid solubilities of Si in α-Ti and β-Ti, Ti_5_Si_3_ tends to precipitate on the α/β phase boundary [20]. Thus, an irregular network architecture is formed [21]. TiB is formed through a eutectic process. A low content of elemental B leads to a typical network architecture [22], mimicking grain boundary structure. This architecture was demonstrated as an effective approach for simultaneously strengthening and toughening. However, the added boron (B) content requires precise control [23]. An appropriate B content in the titanium matrix can achieve an optimal balance between strength and elongation [24], whereas excessive B introduction may lead to a significant strength–ductility trade-off [25]. Therefore, additive manufacturing techniques present a promising potential to tailor the distribution of refinement in TMCs [26].

Conventional manufacturing processes, such as casting [27], forging [28], and powder metallurgy [29], are commonly employed to fabricate in situ TMCs. However, these conventional methods generally require costly and time-consuming mold design. Applying milling on the metal billet to prepare complex-shaped components implies a significant waste of expensive titanium alloys. In contrast, additive manufacturing technology presents a good potential to improve material utilization and reduce time consumption. This will expand the application of titanium alloys [30,31].

Selective laser melting (SLM) is regarded as one of the most promising additive manufacturing technologies. It is a feasible way to produce a near-net-shape complex-shape component with high precision [32,33]. Furthermore, it is convenient for the in situ synthesis of novel materials. During the SLM process, the cooling rate reaches 10^6^–10^8^ K/s, and the materials experience rapid melting and solidification layer-by-layer [34,35,36]. Therefore, this leads to refined and controllable microstructures via adjusting the process parameters [37,38]. This provides a significant potential for producing high-performance metal matrix composite components.

However, SLM is a complex physical process. The key factors, i.e., power energy density and scanning speed, strongly affect intricate dynamic flow behaviors in the molten pool, including convection, turbulence, vaporization, and surface tension effects [39,40]. Introducing reinforcement may further complicate these effects during TMC fabrication. Consequently, inappropriate process parameters during SLM can readily induce defects such as pores and cracks. More importantly, research on SLM-processed TC4-B-Si composites remains extremely limited.

Therefore, this work focused on in situ TC4-B-Si composites. The effects of process parameters on relative density and microstructure were systematically investigated. The microstructure evolution and reinforcement formation mechanisms in the TC4-B-Si system were elucidated. Furthermore, the influence of microstructural features on microhardness was investigated. The relationship between SLM process parameters, microstructural features, and hardness was established. This work expands the material systems for SLM-fabricated TMCs and provides valuable references for producing components with controllable structural characteristics and superior comprehensive performance.

## 2. Experimental

### 2.1. Preparation of Composite Powder

**Figure 1:**
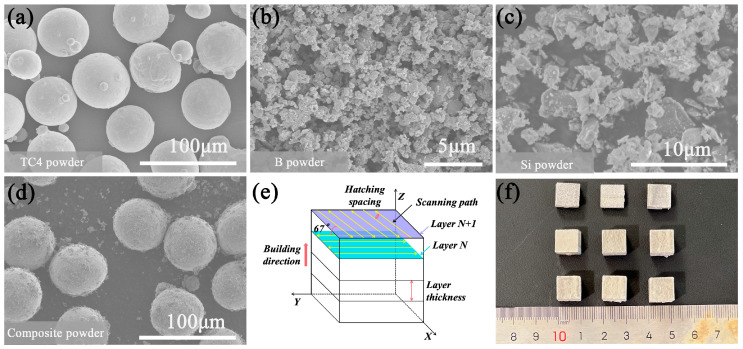
Morphology of powders, printing strategy, and samples: (**a**) TC4; (**b**) B; (**c**) Si; (**d**) Mixture powders; (**e**) Schematic illustration of laser scanning strategy; (**f**) SLM-fabricated TMC samples.

In this work, gas-atomized spherical TC4 powder (50–75 μm, Figure 1a), B powder (99% purity, 100–200 nm average size, Figure 1b), and Si powder (99% purity, 1–5 μm, Figure 1c) were employed. The specified chemical composition of TC4 is presented in Table 1. The target volume fractions of reinforcement were 5 vol.% TiB and 2.5 vol.% Ti_5_Si_3_. Based on the stoichiometric ratios of the reactions B + Ti → TiB and Si + Ti → Ti_5_Si_3_, the calculated additions were 0.94 wt.% B powder and 0.63 wt.% Si powder, respectively. The TC4, B, and Si powders were mixed via a low-energy ball milling process with a ball-to-powder ratio of 2:1, rotation speed of 200 rpm, and milling duration of 4 h. The mixture powder exhibits a relatively uniform distribution of B and Si particles, which adhere to the surfaces of the coarse TC4 particles. The milled TC4 powder is still spherical. This morphology indicates that the mixture powder maintains acceptable flowability and demonstrates high processability for SLM applications.

### 2.2. SLM Process

The SLM process was performed using an E-plus M260 system (E-plus, Hangzhou, China) equipped with a 500 W Yb-fiber laser featuring a beam spot size of 100 μm. The samples were fabricated with a constant layer thickness of 50 μm and a hatch spacing of 120 μm. The laser scanning direction was rotated by 67° between consecutive layers (Figure 1e), and the substrate was preheated to 200 °C. During the SLM process, the powder absorbs laser energy until reaching melting temperature. Consequently, the forming quality in SLM is intrinsically related to the laser power input. To evaluate the influence of process parameters on sample density, we focused on the volume energy density (*E*) serving as a key factor. The values of *E* were determined by the formula [37]

(1) $$E=\frac{P}{v\cdot t\cdot h}\;$$

where *E* represents the volume energy density (J/mm^3^), *P* denotes the laser power (W), *v* is the scanning speed (mm/s), *t* refers to the powder layer thickness (mm), and *h* stands for the hatch spacing (mm). To systematically investigate the effect of volumetric energy density on the forming quality and relative density of the composites, nine composite cubic specimens were fabricated (10 × 10 × 10 mm^3^, Figure 1f) using the SLM processing parameters detailed in Table 2.

### 2.3. Microstructure Characterization and Microhardness

The SLM-ed TC4-B-Si composite samples were separated from the titanium substrate using wire electrical discharge machining (EDM), followed by ultrasonic cleaning in ethanol and drying prior to subsequent characterization. The relative density was determined via Archimedes’ principle, employing theoretical density values of 4.44 g/cm^3^ for TC4 and 4.50 g/cm^3^ for TiB in the calculations. Phase identification was conducted using X-ray diffraction (XRD, Bruker D8 ADVANCE, Billerica, MA, USA) with Cu Kα radiation. For microstructural examination, samples were prepared according to standard metallographic procedures, with both transverse (XOY) and longitudinal (XOZ) sections being ground and polished. The polished specimens were then chemically etched using a solution comprising 3% HF, 5% HNO_3_, and 92% distilled water, and subsequently examined using an optical microscope (OM, Veiyee WY-G, Laizhou, China) and a scanning electron microscope (SEM, Hitachi SU8600, Tokyo, Japan) with energy dispersive spectroscopy (EDS). Microhardness measurements were performed on all nine sample groups using a digital microhardness tester (Veiyee HV-1000STA, Laizhou, China) with a 200 gf load applied for 15 s. To ensure measurement reliability, fifteen indentations were made on each specimen. Then, the highest and lowest values were discarded, and the remaining thirteen measurements were averaged to determine the specimen’s representative microhardness value.

## 3. Result and Discussion

### 3.1. Densification Behavior

**Figure 2:**
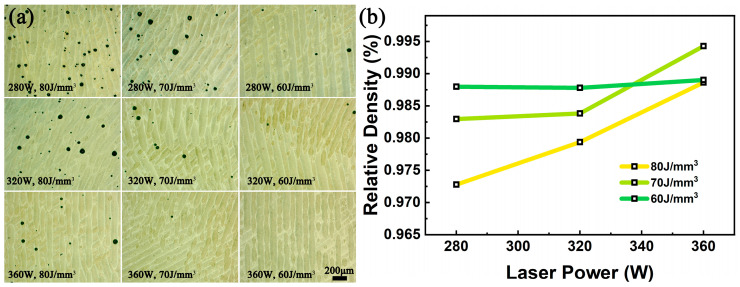
Analysis of sample formability: (**a**) Optical micrographs of SLM-ed TC4-B-Si samples on the cross-sectional surface; (**b**) Relationship between relative density and laser power for TMCs at various scanning speeds.

Figure 2a presents the optical micrographs of the cross-sectional surfaces (XOY) for the SLM-ed TC4-B-Si composites. The pore area fraction was quantitatively measured using Image J 2.14.0 software (Table S1). It can be found that the number of pores decreases from the top-left to bottom-right regions, which is consistent with macroscopic observations. The specimens fabricated at 280 W laser power with 80 J/mm^3^ energy density shows the highest pore concentration, with a porosity of 1.92%. The pore quantity significantly reduces, with the energy density decreasing to 70 J/mm^3^ and further to 60 J/mm^3^. This inverse relationship between energy density and porosity also occurs in the 320 W/360 W samples. It is noted that the number of pores gradually decreases, with the laser power increasing from 280 W to 360 W.

It is remarkable that the pores are all spherically shaped rather than irregular ones. This phenomenon indicates that the power energy density (*E*) was high enough to completely melt the powder. This is significant because insufficient *E* typically leads to incomplete powder melting and unstable molten pools, resulting in irregular lack-of-fusion pores [41]. With various values of *E*, the characteristic spherical pore morphology implies that gas porosity and keyhole-induced pores were the dominant defect formation mechanisms [26].

The relative density of samples measured by Archimedes’ principle is presented in Figure 2b, showing consistent trends with the Image J analysis results. At constant energy density, the relative density of TMCs increases with the increasing laser power. It can be found from Formula (1) that the scanning speed also increases proportionally. Taking *E* as 80 J/mm^3^ as an example (yellow line), sufficient laser power ensures complete metal melting, while higher scanning speeds prevent excessive laser interaction time with the molten pool, thus avoiding keyhole formation. Conversely, when decreasing the scanning speed with a fixed laser power, *E* increases (60 J/mm^3^ to 80 J/mm^3^). Thus, the material relative density reduces. This inverse relationship is induced by excessive energy input (achieved through low scanning speeds), causing aluminum element to evaporate [42]. This promotes keyhole generation, which ultimately decreases composite density. Interestingly, at 360 W laser power, the sample fabricated at 70 J/mm^3^ exhibits higher density than that at 60 J/mm^3^. This is an anomaly due to the scanning speed at *E* of 60 J/mm^3^ being too high. This leads to pool instability and, thus, the generation of numerous micropores.

### 3.2. Phase Identification

**Figure 3:**
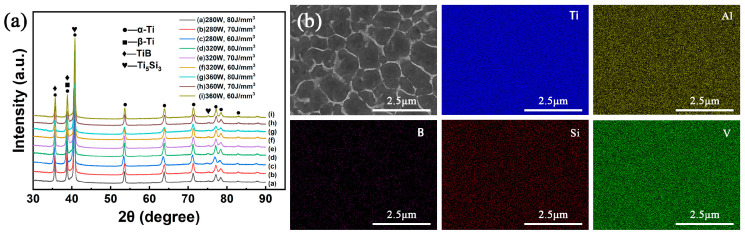
The phase and composition identification: (**a**) XRD patterns of the as-built TC4-B-Si composites with different parameters; (**b**) EDS images showing the cross-section of the as-built TC4-B-Si composites.

In the TC4-B-Si system, in situ reacted reinforcement is generated during phase transition. To investigate this issue, the phase composition and distribution analyses were conducted. Figure 3a presents the XRD pattern comparison between the nine composite samples. It is shown that TMCs have the same phase compositions, i.e., α-Ti, β-Ti, TiB, and Ti_5_Si_3_. It is noted that the B and Si diffraction peaks are absent in the XRD patterns. This implies that B and Si raw powders were completely dissolved in the molten pool. This promotes in situ reactions between Ti and B/Si to form TiB and Ti_5_Si_3_ reinforcements during solidification. In addition, the full width at half maximum (FWHM) of the predominant peak was measured to compare the grain size of TMCs, which is analyzed specifically in Section 3.3.

Due to the relatively low content of TiB and Ti_5_Si_3_ phases, the corresponding diffraction peaks are relatively weak. Thus, a detailed investigation of reinforcement in TMCs is required. Here, EDS tests were performed on the cross-section (XOY) of the sample with 280 W, 60 J/mm^3^ (Figure 3b). The mapping shows an interconnected network morphology. The boron distribution, represented by the purple signal, shows significant enrichment in the bright network-structured regions (Figure S1). Due to the extremely limited solubility of boron in both α-Ti and β-Ti phases, it preferentially reacts with titanium to form TiB through eutectic transformation. Based on the above description, it can be clearly determined that the bright phase is TiB reinforcement, while the gray phase is TC4 matrix. Remarkably, rarely significant Si enrichment or segregation was detected by EDS. This observation reveals that the Ti_5_Si_3_ phases are dispersed in the matrix at a scale below the EDS detection limit. The XRD pattern depicts a clear Ti_5_Si_3_ peak. Therefore, the Ti_5_Si_3_ particles may be nano-scale. On the other hand, some Si atoms may exist in a solid solution form uniformly distributed within the Ti matrix. This phenomenon can be primarily attributed to the characteristic ultra-rapid solidification kinetics of the SLM process, which typically achieves cooling rates ranging from 10^6^ to 10^8^ K/s. Such extreme cooling conditions effectively suppress the diffusion-controlled precipitation of Ti_5_Si_3_ phases, resulting in the supersaturated solid solute Si element in the Ti matrix.

### 3.3. Microstructure Characterization

**Figure 4:**
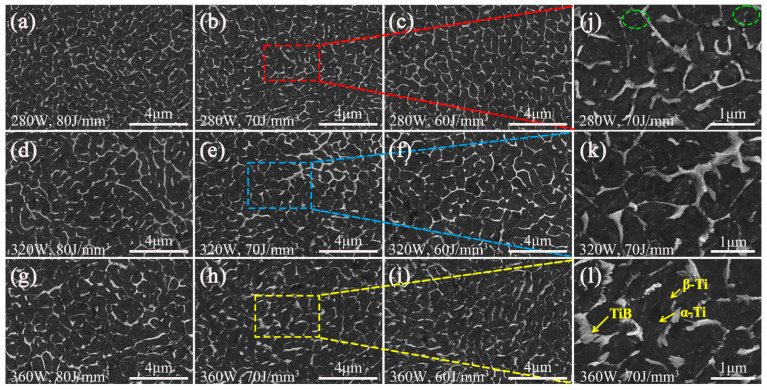
Cross-section microstructures (secondary electron images) of the TC4-B-Si composites with various energy densities, i.e., (**a**,**d**,**g**) 80 J/mm^3^, (**b**,**e**,**h**) 70 J/mm^3^, (**c**,**f**,**i**) 60 J/mm^3^, (**j**–**l**) High-magnification SEM image at an energy density of 70 J/mm^3^, with three laser powers: (**a**–**c**,**j**) 280 W; (**d**–**f**,**k**) 320 W; (**g**–**i**,**l**) 360 W.

Figure 4 displays the microstructures in the transverse (XOY) cross-section. It can be observed that TiB reinforcement preferentially grows along the TC4 matrix grain boundary. During the laser melting process, the TiB phase formation consumes most of the B element. It is shown that the TC4 matrix is composed of α and β phases, and TiB is mainly whisker-like shaped (Figure 4j–l). The TiB whiskers range as a fence on the network boundary. In the reticular cell, the nano β-Ti plates are between α-Ti banded grains (Figure S2). In this process, TiB preferentially grows along the [010] crystallographic direction [43]. The mono-reinforcement presents individual whisker or the clustering of parallel whiskers in close contact. Thus, a colony structure is formed. Additionally, some plate-like TiB phases (circled in Figure 4j) can be observed. This morphology is attributed to the extremely high cooling rate, meaning that partial B element is supersaturated solid-dissolved in β-Ti. Subsequently, the plate-like TiB precipitated through solid phase transformation, due to the precipitation behavior being dominated by surface energy rather than strain energy to satisfy the minimum energy principle [44].

It is noted that the network distributed TiB exhibits submicron-scale or even nano-scale structure. For Figure 4a–c, the corresponding FWHM values were measured, i.e., 0.307, 0.344, and 0.379, respectively. This means that, as the energy density decreasing from 80 J/mm^3^ to 60 J/mm^3^, the network cell size gradually refines. This is attributed to the presence of boron and the ultra-rapid cooling of the SLM process. B and Si elements act as heterogeneous nucleation sites, significantly enhancing the nucleation rate of primary β-Ti grains. During the SLM process, the cooling rate of the molten pool could reach an extremely high value (10^6^–10^8^ K/s), shortening the molten pool life and inhibiting the growth of primary β-Ti grains. Consequently, the primary β-Ti grains are fined, and thus the TiB network cell is also fined.

With decreasing energy density, the microstructure becomes increasingly heterogeneous (Figure 4d–f). With a high energy density (80 J/mm^3^), a homogeneous network structure was formed (Figure 4d). When the energy density decreases to 70 J/mm^3^ and 60 J/mm^3^, the bright network structure becomes inhomogeneous (Figure 4e,f). This phenomenon occurs because a higher laser scanning speed increases the cooling rate of the molten pool, thereby shortening the interaction time between the laser heat source and the powder bed. Thus, the molten pool convection behavior was weakened. Consequently, B atoms distribute unevenly, leading to localized B enrichment and the clustering of TiB particles in specific regions, ultimately resulting in microstructural heterogeneity [41].

**Figure 5:**
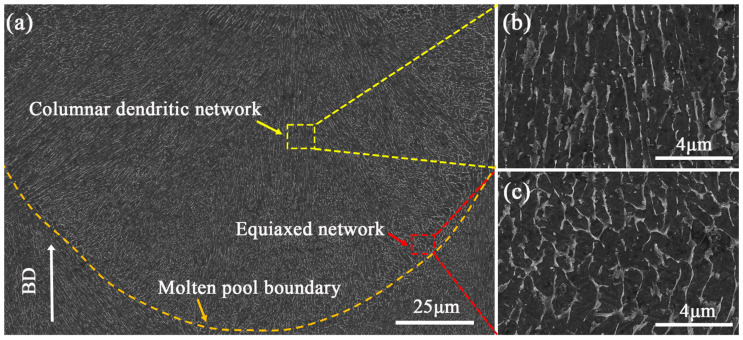
Micrographs (secondary electron images) showing the microstructures of the TC4-B-Si composites on the longitudinal section: (**a**) a molten pool; (**b**) dendrite (columnar) β grains at the top of the molten pool; (**c**) equiaxed β grains at the bottom of the molten pool.

Furthermore, to thoroughly investigate the microstructure of longitudinal sections (XOZ), columnar grains in a single molten pool were observed by SEM (Figure 5a). During laser irradiation, the mixture powders rapidly melt to form a molten pool. In this process, the B and Si powders were dissolved in the molten pool. The instantaneous high-energy input and intense heat accumulation leads to extremely high undercooling at the molten pool bottom. This phenomenon results in the extensive nucleation of primary β-Ti grains. Thus, fine equiaxed grains were formed (Figure 5c). As the bottom of the molten pool solidifies to form a thermal resistance layer, the heat dissipation direction tends to be uniform. As such, a huge temperature gradient perpendicular to the molten pool boundary was generated. Subsequently, the temperature gradient promotes β-Ti grain to grow further towards the center of the molten pool along the direction of the heat flow. Therefore, the Ti grain grew with a strong preferred direction to form a columnar morphology (Figure 5b).

**Figure 6:**
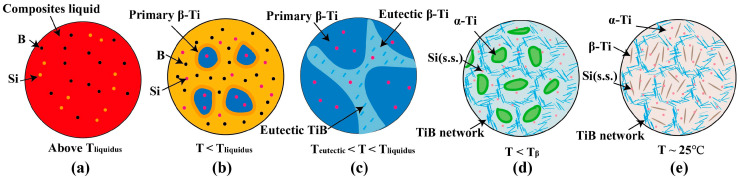
Schematic illustration of the microstructure evolution: (**a**) TC4, B, and Si are melted into the molten pool above the liquidus temperature; (**b**) primary β-Ti nucleates with the temperature below the liquidus; (**c**) eutectic (TiB + β-Ti) forms at the eutectic temperature; (**d**) β-Ti transforms into α-Ti, Si remains supersaturated dissolved in the matrix; (**e**) the final microstructure at room temperature.

As previously mentioned, the SLM-ed TMC samples contained an average boron content of 0.94 wt.%. According to the Ti–B binary phase diagram [44], the composite system is in the hypoeutectic region. The solidification and phase transformation sequence proceeds as follows: Liquid → Liquid + primary β-Ti → primary β-Ti + eutectic (TiB + β-Ti) → TiB + β-Ti + α-Ti. During hypoeutectic solidification, primary β-Ti grain nucleates and grows between the liquidus and eutectic transformation temperatures (Figure 6a,b). When the temperature decreases below the eutectic line, the remaining molten liquid experiences a eutectic process, forming a mixture of β-Ti and TiB (Figure 6c). Further cooling induced an allotropic transformation. Below the β-transus temperature, β-Ti grains transform to α-Ti. The Si element remains supersaturated dissolved in the matrix due to the extremely high cooling rate (Figure 6d).

The extremely low solid solubility of boron in titanium causes its rejection from primary β-Ti nuclei into the molten pool. This implies an extremely low solid solubility of B element in primary β-Ti grains. Thus, most boron element was ultimately applied to form TiB phase. This solute partitioning phenomenon led to boron enrichment in the liquid ahead of the solidification front. As solidification progresses, the boron accumulation intensifies constitutional supercooling. This behavior decreases the stability of the liquid/solid interface. Therefore, this provides a driving force for the nucleation of finer Ti grains ahead of the solidification front. Furthermore, the growth of existing Ti nuclei is constrained by boron element segregation at the liquid/solid interface. This is beneficial to refine the Ti grains. The observed nano-scale network architecture and grain refinement phenomena (Figure 4a–c) are direct results of these solidification mechanisms.

### 3.4. Microhardness

**Figure 7:**
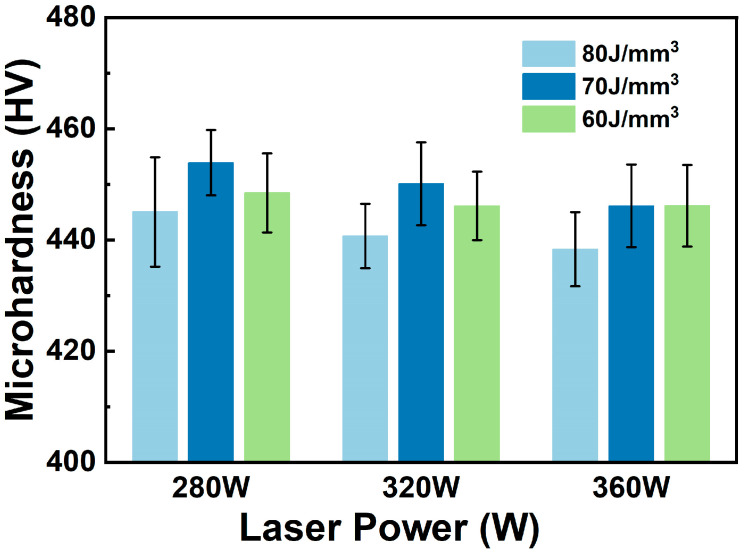
Vickers microhardness of the TC4-B-Si composites with various values of laser power and energy density.

Figure 7 illustrates the microhardness of TC4-B-Si composite fabricated with various process parameters. Compared with TC4 alloy (354 HV), the microhardness of TMCs (438–454 HV) is significantly enhanced. The rate of increase is 23.7–28.2%. From the viewport of hardness, the preferred power energy density is maybe 70 J/mm^3^. From Figure 2a, the specimen with lower laser power exhibits higher density, for which microhardness is also higher (Figure 7). Therefore, the preferred laser power is maybe 280 W (or even lower).

Generally, microhardness directly correlates with the density of the test sample. In other words, higher density typically implies greater resistance to plastic deformation and consequently higher microhardness. Yet, because the density of all samples exceeds 97%, the density effect is weak. For a metal matrix composite, the other factors also strongly affect the hardness, i.e., the grain size and the reinforcement distribution. With the energy density decreasing to 70 J/mm^3^, the fine grain strengthening effect leads to higher microhardness. However, as the energy density further reduces to 60 J/mm^3^, the heterogeneous distribution of TiB is more significant. This structure leads to a local coarsening of the TiB phase. The coarse TiB phase is prior to fracture under load. Thus, the load-bearing capacity of reinforcement is reduced. With an energy density of 60 J/mm^3^, the weakening effect of the coarsening of the TiB phase suppresses the strengthening effect of grain refinement. Therefore, the specimen with laser power equal to 280 W and energy density set at 70 J/mm^3^ shows the highest microhardness (454 HV). On the other hand, with laser power increasing from 280 W to 360 W, the microhardness changes slightly.

Based on the near-net forming advantage of SLM, the optimal processing window for TMCs was efficiently identified and the “process–structure–performance” relationship of the TC4-B-Si composite system was established. The fabricated TMCs exhibit an ultrafine multi-scale network structure, which significantly enhances the mechanical properties of titanium matrix. In addition, the content of the reinforcing phase has a crucial impact on the microstructure [45,46,47], which is the direction that we need to consider in the future. While the current study focuses on microstructure and microhardness due to the small sample size (10 mm cubes) used in process optimization, future work will include larger specimens to evaluate tensile strength and fracture toughness for industrial-grade validation. The advanced material presents great application potential in lightweight and wear-resistant structural components in various industries, including rail transportation, biomedical implants, and intelligent robotics.

## 4. Conclusions

In summary, TC4-B-Si composites were successfully fabricated via SLM, with various process parameters. It was found that increasing the laser power and reducing the energy density is a feasible way to decrease the content of pores. The density of all composite samples exceeds 97%. This means that the TC4-B-Si composite system has a good formability. Thus, the influence of the density effect on microhardness is relatively weak. The TC4 matrix consists of α and β phases, and the in situ formed TiB phase mainly exhibits a whisker-like morphology, which arranges regularly as a fence on the grain boundaries of primary β-Ti grains, forming a network structure. In addition, most Si atoms are supersaturated solid-dissolved in the titanium matrix. The XRD results indicate that a small amount of nano-scale Ti_5_Si_3_ exists. An investigation of process parameters presents that the grain size decreases with reduced energy density. This is because the cooling rate increased significantly. As such, the fine grain strengthening effect consequently leads to a high microhardness. With further reduction in energy density, the cooling rate increases and the TiB phase distribution becomes increasingly heterogeneous. This results in a localized coarsening of the TiB phase. Thus, the load transfers from matrix to reinforcement ineffectively. This phenomenon leads to a decreasing microhardness. The ideal process parameters, i.e., laser power of 280 W and energy density of 70 J/mm^3^, keep a good balance between these two factors. Therefore, the microhardness reaches a maximum value of 454 HV, which presents an enhancement of 28.2% compared with TC4 alloy.

## Figures and Tables

**Table 1:** Specified chemical composition of TC4.

Powder	Mass Fraction (wt.%)							
TC4	Ti	Al	V	Fe	C	H	O	N
	Bal.	6.13	4	0.3	0.08	0.012	0.09	0.016

**Table 2:** SLM process parameters.

Sample Number	Laser Power (W)	Laser Scanning Speed (mm/s)	Layer Thickness (mm)	Scan Track Spacing (mm)	Laser Energy Density (J/mm^3^)
1	280	583	0.05	0.12	80
2	280	667	0.05	0.12	70
3	280	778	0.05	0.12	60
4	320	667	0.05	0.12	80
5	320	762	0.05	0.12	70
6	320	889	0.05	0.12	60
7	360	750	0.05	0.12	80
8	360	857	0.05	0.12	70
9	360	1000	0.05	0.12	60

## Data Availability

Data are contained within the article.

## Supplementary Materials

The following supporting information can be downloaded at: https://www.mdpi.com/article/10.3390/ma18184223/s1, Table S1 Pore area fraction of SLM-ed TMCs; Figure S1: TiB network architecture: (a) High-magnification SEM image; (b) SEM-EDS maps of element B; Figure S2: High-magnification SEM image of phases.
