# Supplementary material for: Effect of Ultra-Rapid Heating/Cooling on the Microstructure and Properties of TC4-B-Si Titanium Matrix Composites
Source: Materials (Basel). 2025 Sep 9;18(18):4223. doi: 10.3390/ma18184223 (PMC12471907; doi:10.3390/ma18184223)
Supplement: Supplementary file 1 [file materials-18-04223-s001.zip › materials-3798073-supplementary.pdf]

# Supplementary Materials

## Effect of Ultra-Rapid Heating/Cooling on the Microstructure and Properties of TC4-B-Si Titanium Matrix Composites

Xiaonan Lu [1,2], Jianchao Li [2], Cheng Liu [2], Likun Wang [1], Sainan Ma [1], Bo Yuan [4], Bowen Gong [2], Wenting Ouyang [2], Huan Wang [2], Xiang Gao [1,2*], Huiping Tang [3], and Hua-Xin Peng [1,2*]

[1] Ningbo Global Innovation Center, Zhejiang University, Ningbo 315100, China

[2] Institute for Composites Science Innovation (InCSI), School of Materials Science and Engineering, Zhejiang University, Hangzhou 300027, China

[3] Advanced Materials Additive Manufacturing Innovation Research Center, Hangzhou City University, Hangzhou 310015, China

[4] Institute of Intelligent Manufacturing Technology, ShenZhen Polytechnic University, ShenZhen 518055, China

*Corresponding author. E-mail address: hxpengwork@zju.edu.cn (HXP), gaoxiang1986@zju.edu.cn (XG).

The pore area proportion of TMCs prepared by nine groups of printing process parameters was measured by using Image J software, as shown in Table S1.

Table S1 Pore area fraction of SLM-ed TMCs

| Parameters | Area fraction of pores (%) | | |
| --- | --- | --- | --- |
| Energy density / Laser Power | 80 J/mm$^3$ | 70 J/mm$^3$ | 60 J/mm$^3$ |
| 280W | 1.915638721 | 1.501867466 | 0.216771172 |
| 320W | 1.324544279 | 0.551132466 | 0.142278301 |
| 360W | 0.687302973 | 0.114120751 | 0.027095326 |

The high-magnification SEM image of the TiB network architecture (Figure S1a) and the corresponding B element distribution map (Figure S1b) are listed separately for clearer observation.

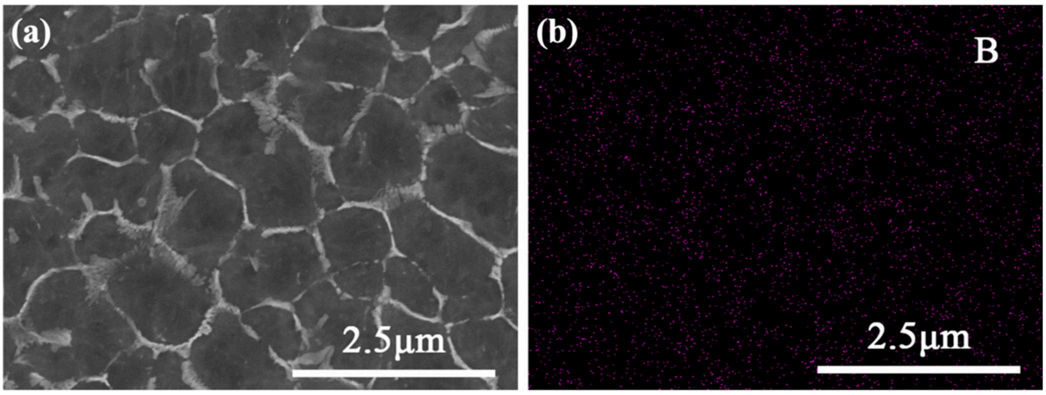


Figure S1. TiB network architecture: (a) High-magnification SEM image; (b) SEM-EDS maps of element B.

In order to distinguish the TC4 matrix phase and the TiB whiskers more clearly, the high-magnification SEM image is shown in Figure S2.

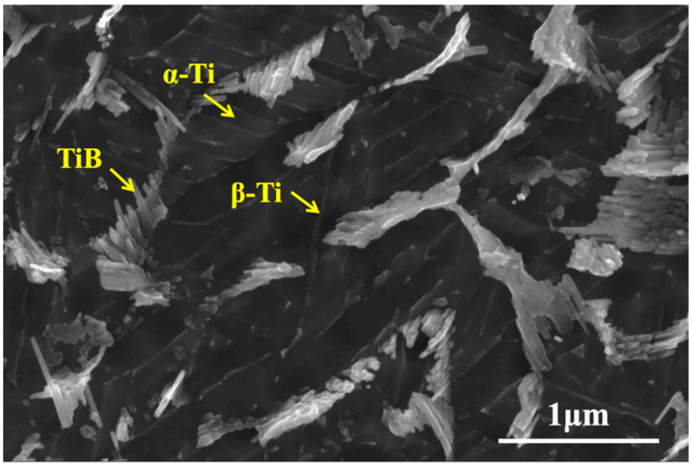


Figure S2. High-magnification SEM image of phases.
